# Chromothripsis in Chronic Lymphocytic Leukemia: A Driving Force of Genome Instability

**DOI:** 10.3389/fonc.2021.771664

**Published:** 2021-11-26

**Authors:** Kristyna Zavacka, Karla Plevova

**Affiliations:** ^1^ Department of Internal Medicine - Hematology and Oncology, University Hospital Brno & Faculty of Medicine, Masaryk University, Brno, Czechia; ^2^ Center of Molecular Medicine, Central European Institute of Technology, Masaryk University, Brno, Czechia; ^3^ Institute of Medical Genetics and Genomics, University Hospital Brno & Masaryk University, Brno, Czechia

**Keywords:** chromothripsis, chronic lymphocytic leukemia, complex chromosomal rearrangements, copy number alterations, genomic array, paired-end sequencing, oncogene amplification, tumor suppressor inactivation

## Abstract

Chromothripsis represents a mechanism of massive chromosome shattering and reassembly leading to the formation of derivative chromosomes with abnormal functions and expression. It has been observed in many cancer types, importantly, including chronic lymphocytic leukemia (CLL). Due to the associated chromosomal rearrangements, it has a significant impact on the pathophysiology of the disease. Recent studies have suggested that chromothripsis may be more common than initially inferred, especially in CLL cases with adverse clinical outcome. Here, we review the main features of chromothripsis, the challenges of its assessment, and the potential benefit of its detection. We summarize recent findings of chromothripsis occurrence across hematological malignancies and address its causes and consequences in the context of CLL clinical features, as well as chromothripsis-related molecular abnormalities described in published CLL studies. Furthermore, we discuss the use of the current knowledge about genome functions associated with chromothripsis in the optimization of treatment strategies in CLL.

## Introduction

Chronic lymphocytic leukemia (CLL) is the most common adult leukemia in Western countries with a highly variable clinical course. Several recurrent chromosomal alterations have been associated with prognosis and may guide risk-adapted therapy. Besides deletions on chromosomes 11, 13, 17, and trisomy 12, high genomic complexity (high-GC) has also been recognized as a feature with prognostic value (1, 2) and is associated with poor clinical outcome (3, 4). Cytogenetics and array-based methods define high-GC as five or more chromosomal defects (1, 2). In many instances, highly complex karyotypes can be caused by chromothripsis (cth) (5), a genomic event by which a single or a limited number of chromosomes are shattered into pieces, followed by error-prone reassembly (6–9).

Among all cancers, it was CLL where the evidence of cth was reported for the first time. This finding was made already a decade ago *via* the whole-genome sequencing screening of 10 CLL patients (5). In a sample from a 62-year-old woman without any previous CLL treatment, a massive rearrangement of chromosomal arm 4q and focal alterations on chromosomes 1, 12, and 15 were found, showing striking patterns. It was proved that this complex genomic remodeling had occurred before the diagnosis and persisted until the rapid disease relapse after alemtuzumab treatment without further evolution. The phenomenon was termed chromothripsis (from Greek; chromos for chromosome, thripsis for shattering into pieces) and was subsequently observed in many other tumor types (5, 10–15).

**Figure 1:**
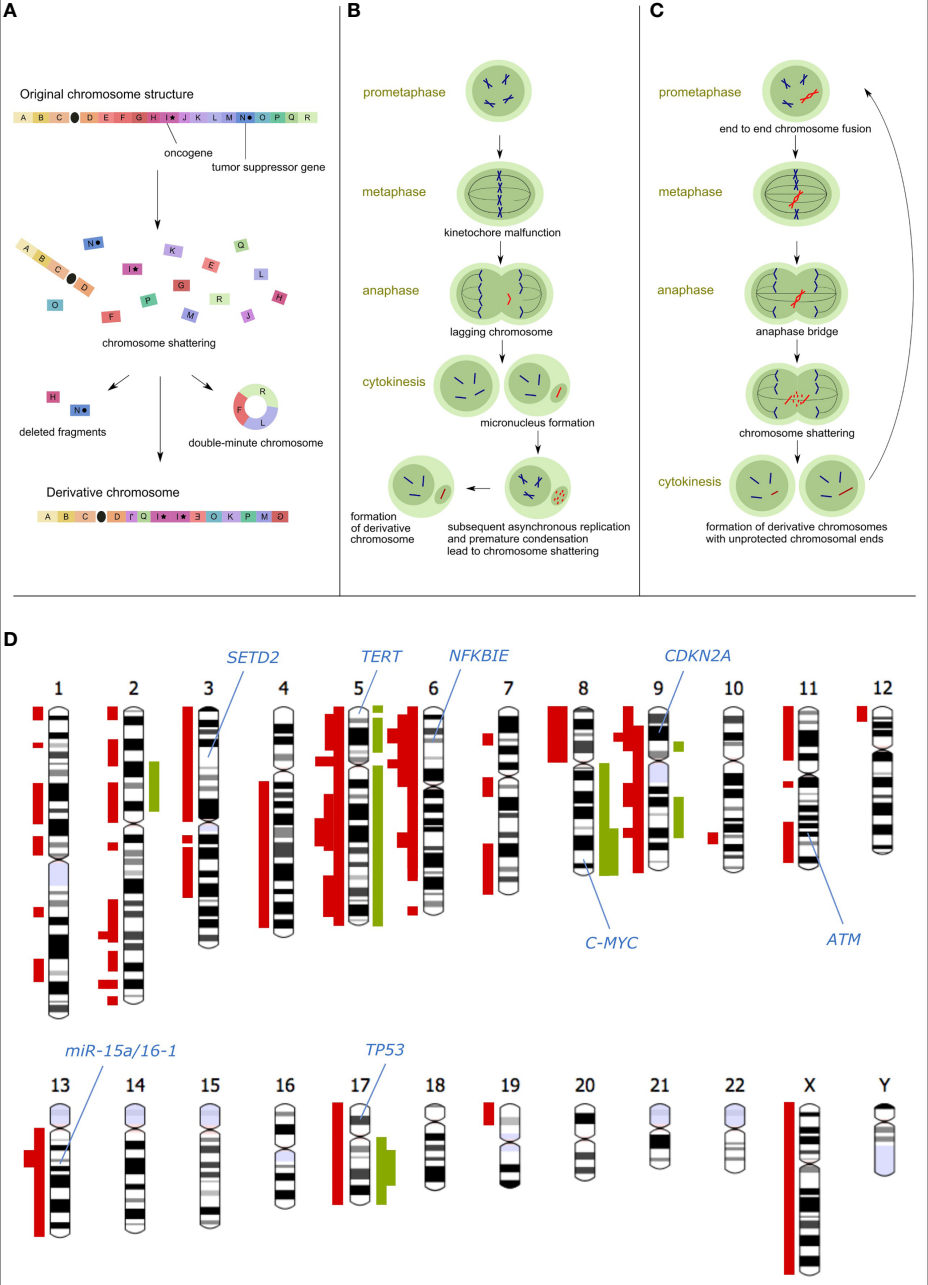
Causes and consequences of chromothripsis. **(A)** Schematic model of chromosome shattering and reassembly *via* cth: After chromosome fragmentation, some regions are incorporated (possibly in multiple copies) into a derivative chromosome, whereas other regions can be lost or fused to episomal structures called double-minute chromosomes. **(B)** The micronuclei hypothesis of the cth origin: Chromosomes that are missegregated during cell division are entrapped in the micronucleus, followed by asynchronous replication compared to the main nucleus. This leads to premature chromosome condensation and shattering. Rejoining of fragments gives rise to the derivative chromosome which can subsequently be reincorporated into the main nucleus. **(C)** The origin of cth due to breakage-fusion-bridge (BFB) cycles and telomere crisis: Chromosome ends that become unprotected due to telomere shortening are fused into a dicentric chromosome containing two centromeres. In the subsequent cell cycle, this unstable structure is pulled to opposite spindle poles forming an anaphase bridge between the two daughter cells. The rupturing bridge generates two new unprotected chromosomal ends and initiates a new round of the BFB cycle. This repeats until the derivative chromosome becomes stable. **(D)** Chromosomal ideograms with cth-derived gains (green) and losses (red) observed in the following CLL studies: Stephens et al., 2011 (5); Edelmann et al., 2012 (3); Pei et al., 2012 (16), Bassaganyas et al., 2013 (17); Salaverria et al., 2013 (18); Tan et al., 2015 (19); Parker et al., 2016 (20); Leeksma et al., 2021 (2). The thickness of the highlighted loci corresponds to the number of studies referring to the respective regions affected by cth. Only studies mentioning specific affected areas and distinguishing individual patients were compiled.

In contrast to the traditional view of tumorigenesis as the multi-step accumulation of mutations, cth arises *via* a single devastating event. Within a single cell division, tens to hundreds of DNA double-strand breaks are generated and imperfectly assembled into derivative chromosomes, most often *via* non-homologous end joining (NHEJ), whereas some fragments can be lost (**Figure 1A**). The massively rearranged genomes of the cells that survive such an event propagate in daughter clones and are likely to have gained a strong selection advantage, as cth could disrupt the functions of tumor suppressors, support the oncogene amplification, and/or give rise to pathogenic gene fusions. Thus, cth is a potential driving force of malignant transformation and tumor progression.

## Detection of Chromothripsis-Like Patterns

Cth is characterized by several hallmarks that set it apart from other complex genomic changes: (a) occurrence of tens to hundreds of chromosomal rearrangements with pronounced clustering, (b) random orientation of rearrangements resulting in equal representation of deletions, inversions, and tandem duplications, (c) copy-number alterations (CNAs) oscillating between two (occasionally three) copy-number states, (d) alterations of segments that retained heterozygosity and segments with loss-of-heterozygosity (LOH), (e) structural rearrangements displaying a bias toward occurring on a single chromosome homolog, and (f) presence of double-minute chromosomes (5, 15). The evidence of different cth patterns in various cancer types and among individual cases (21) suggests different mechanisms of its origin. The mechanisms, presumed most frequently to cause cth, include asynchronous DNA replication in abnormal nuclear structures called micronuclei (6, 22, 22) (**Figure 1B**) and the fragmentation of dicentric chromosomes resulting from the telomere crisis due to their extreme shortening (23) (**Figure 1C**).

Since the genomic profile originating in cth could be similar to stepwise processes, the detection of cth is often challenging. Therefore, a set of criteria was generated for accurate and reproducible cth inference (7). Most of these criteria take into account the entire set of structural rearrangements that occurred on a chromosome, including the relative order and orientation of rearranged segments. They are typically detected using whole-genome paired-end DNA sequencing. Copy-number states can also be analyzed by array-based comparative genomic hybridization (aCGH) or single nucleotide polymorphism (SNP) arrays. However, for the most accurate detection of cth, it is desirable to use a complex approach that combines sequencing genomic methods with molecular cytogenetics and other complementary methods (24). A conventional karyotyping of metaphases can be useful to identify numerical and structural chromosomal abnormalities. Various fluorescence *in situ* hybridization (FISH) techniques may aid the identification of interacting chromosome partners and localization of breakpoints. Spectral karyotyping in combination with fluorescent locus-specific probes can effectively detect the double-minute chromosomes (5). Above that, RNA-Seq can assist in revealing abnormalities at the transcriptional level such as *de novo* fusion transcripts or abnormal gene expression, both of which can be revealed with advanced analytical methods (25).

## Chromothripsis in Hematological Malignancies

Cth has been observed in primary tumors of various histological types, including hematological malignancies, such as lymphomas (19, 21, 26), multiple myeloma (11, 26, 27), myelodysplastic syndrome (26, 28, 29), and leukemias (2, 3, 5, 15–21, 24, 30–39). The prevalence of cth across cancer types ranges from units to tens of percent with the highest proportions in sarcomas – up to 100% (5, 21, 40). However, the comparison of published studies provides only rough estimation due to different methodologies and definitions used for cth scoring. The cth frequencies observed in hematological malignancies are summarized in **Table 1**.

**Table 1:** Prevalence of chromothripsis in CLL and other hematological malignancies.

Reference	Clinical characterization of the cohort	Clinical characterization of cth cases	n/N	Cth prevalence	Method
**Chronic lymphocytic leukemia**					
Stephens et al., 2011 (5)	not specified	rapid relapse after alemtuzumab	1/10	**10%**	WGS
Edelmann et al., 2012 (3)	treatment-naïve; samples from the GCLLSG CLL8 trial	poor survival; 74% with unmutated IGHV; 32% with mutated *TP53*	19/353	**5.4%**	SNP array
Salaverria et al., 2015 (18)	26% treatment-naïve	poor survival; 75% with *TP53* abnormality (mutation and/or deletion)	8/180	**4.4%**	aCGH
Puente et al., 2015 (38)	treatment-naïve	26% with mutated *TP53*; 26% with inactivated *SETD2*, 25% with loss of *mir-15a/mir-16*	15/452	**3.3%**	SNP array, WGS
Parker et al., 2016 (20)	93% treatment-naïve; 84% of samples from the ADMIRE, ARCTIC, UK CLL4, GCLLSG CLL8, and SCSG CLL2O trials	poor outcome; 26% with *SETD2* deletion	27/1,006	**2.7%**	SNP array
Burns et al., 2018 (39)	52% treatment-naïve	with *TP53* deletion	1/46	**2.2%**	WGS
Cortés-Ciriano et al., 2020 (21)	data from the PCAWG Consortium (41)	not specified	1/86	**1.2%**	WGS
Leeksma et al., 2021 (2)	86% treatment-naïve; samples from 13 CLL diagnostic centers participating in ERIC	poor survival; all with *TP53* abnormality (mutation and/or deletion) and del(11q)	32/2,293	**1.4%**	SNP array, aCGH
Ramos-Campoy et al., 2021 (24)	treatment-naïve; 47% with complex karyotypes	poor outcome, 73% with *TP53* abnormality	30/340	**8.8%**	SNP array, aCGH
**Acute myeloid leukemia**					
Rausch et al., 2012 (15)	non-M3 AML; treatment-naïve; adults	poor survival, 89% with mutated *TP53*	9/108	**8.3%**	SNP array
Fontana et al., 2018 (32)	82% *de novo* AML, 12% AML secondary to myelodysplastic syndrome, 1% AML secondary to myeloid neoplasms, 5% therapy-related AML; mostly adults (median age 59.35)	poor outcome; 70% of cases treated with chemotherapy did not respond; 88% with *TP53* abnormality (mutation and/or deletion)	26/395	**6.6%**	SNP array
**Myelodysplastic syndrome**					
Kim et al., 2013 (26)	data from the GEO database (42)	not specified	7/393	**1.8%**	aCGH
Zemanova et al., 2014 (28)	treatment-naïve; with complex chromosomal rearrangements (≥3 aberrations)	not specified	77/157	**49%**	SNP array
Abáigar et al., 2016 (29)	treatment-naïve	high-risk MDS; all died within one year; all with mutated *TP53*	3/240	**1.3%**	aCGH
**Acute lymphoblastic leukemia**					
Zhang et al., 2012 (35)	childhood early T cell precursor ALL	2 cases relapsed 8 and 13 months after diagnosis, 1 case underwent bone marrow transplantation; all died	3/12	**25%**	WGS
Li et al., 2014 (36)	childhood ALL; 56% with sporadic iAMP21, 44% with rob(15;21)c-associated iAMP21	not specified	8/9	**89%**	WGS
Ratnaparkhe et al., 2017 (37)	childhood ataxia-telangiectasia-related T-ALL	1 case died 2 years after diagnosis, 1 case died from toxicity, 3 cases still alive (2/3 in remission)	5/7	**71%**	WGS
Ratnaparkhe et al., 2017 (37) *	sporadic childhood T-ALL	not specified	4/92	**4.3%**	WGS
**Multiple myeloma**					
Magrangeas et al., 2011 (11)	treatment-naïve	50% with rapid relapse	10/764	**1.3%**	SNP array
Stevens-Kroef et al., 2012 (27)	82% treatment-naïve	not specified	1/28	**3.6%**	SNP array
Kim et al., 2013 (26)	data from the GEO database (42)	not specified	8/391	**2%**	aCGH
Voronina et al., 2020 (43)	data from the NCT/DKTK-MASTER platform (44)	not specified	2/6	**33%**	WGS
**Lymphoma**					
Cortés-Ciriano et al., 2020 (21)	mature B cell non-Hodgkin lymphoma; data from the PCAWG Consortium (41)	not specified	19/105	**18%**	WGS

*refers to unpublished data discussed with Meijerink et al., partly published in Li et al., 2016 (45).

n, the number of cth cases; N, the total number of cases analyzed in the respective study; GCLLSG, German CLL Study Group; PCAWG, Pan-Cancer Analysis of Whole Genomes; ERIC, European Research Initiative on CLL; GEO, Gene Expression Omnibus; NCT/DKTK-MASTER, National Center for Tumor Diseases/German Cancer Consortium-Molecularly Aided Stratification for Tumor Eradication; WGS, whole-genome sequencing; SNP array, single-nucleotide polymorphism array; aCGH, array comparative genomic hybridization.

For most hematological diseases, cth provides independent prognostic information and is associated with adverse clinical outcome. In myelodysplastic syndrome, the complex chromosomal rearrangements caused by cth are related to advanced disease stages prone to transform to acute myeloid leukemia (AML); as a consequence, they recurrently involve 5q deletions (28). Similarly, AML patients with cth have a high recurrence of 5q losses, and also *TP53* dysregulation and the presence of marker chromosomes (30–32). Besides that, cth appears to be mutually exclusive with *FLT3* and *NPM1* mutations (30, 32). In acute lymphoblastic leukemia (ALL), cth occurs predominantly in specific subgroups, such as early T cell precursor ALL (35, 37), iAMP21 B-ALL (36), and ataxia-telangiectasia-related T-ALL (37).

The evidence of cth cases described in CLL indicates that this phenomenon is a recurrent event. By exploring larger cohorts of CLL patients, cth was observed with frequencies from 1.2 to 10% (2, 3, 5, 18, 21, 24, 38, 39). Although the reported prevalence is relatively small, the analysis of cth-like patterns may be beneficial for clinical decision-making and precision medicine, as cth represents a driving force of genome evolution in CLL (5, 16, 17, 20).

## Impact of Chromothripsis on CLL Onset and Progression

CLL patients with cth (cth-CLL) were shown to have inferior time to first treatment (24), progression-free survival (3), and overall survival (2, 3, 18, 38). The overall survival of cth-CLL cases was even worse than of cases with *TP53* abnormality or del(11q) without cth (2). The majority of cth-CLL cases have unmutated IGHV (3, 17, 24, 38, 39). Two studies concluded that cth is more frequent in the IGHV-unmutated group with statistical significance (3, 38). There is also a strong link between the presence of cth and high-risk genomic aberrations like del(11q) and del(17p) (2, 3, 16, 17, 19, 20, 24, 39).

Some studies reported that cth occurs before the CLL diagnosis indicating that the complex genomic remodeling could be a CLL-initiating event (5, 16) or one of the earliest events in the CLL pathogenesis (20). On the contrary, a case study from 2013 showed that cth is not necessarily triggering the CLL onset. In this case, cth was a consequence of previous alterations accumulated since the time of diagnosis and contributed to the increase of CLL aggressiveness, as a subclone carrying complex structural variants expanded and outbalanced the predominant tumor population before the first treatment (17). Interestingly, the cth-subclone was eradicated by chemotherapy and did not reappear throughout a 10-year follow-up period. This observation contrasts other data strongly associating cth-clones with chemotherapy resistance and/or poor clinical outcome (2, 3, 5). That points to the substantial need for larger cohorts of cth cases to be analyzed to better understand the dynamics of cth in CLL.

## Genomic Regions Associated With Chromothripsis in CLL

Chromosomes 2, 3, 6, 8, 9, 11, 13, and 17 were impacted by cth in CLL most frequently (2, 3, 16, 17, 19, 20, 39) (**Figure 1D**). Many cth-CLL cases harbor del(17p) (2, 3, 16, 19, 20, 24, 39) spanning the *TP53* gene, the most important predictor of disease and treatment outcome (46–51). Alterations in *TP53* are the most common changes associated with cth in medulloblastoma (15), acute myeloid leukemia (15), pediatric cancers (52), and CLL (2, 3, 20, 24, 38). *TP53* is responsible for cell cycle control, genome maintenance, and apoptosis (53, 54), confirming its plausible involvement in genome instability preceding cth. The frequent co-occurrence of *TP53* alterations and cth in CLL supports both possibilities of their relation, i.e. cth resulting from *TP53* disruption as well as cth leading to *TP53* abnormalities and therefore more aggressive disease. Alterations in *ATM* including del(11q) and gene mutations can also explain the rise of cth considering its role in the regulation of the DNA damage response (DDR) and were observed in patients with cth (2, 3, 20, 39). In this context, Bassaganyas et al. (17) observed the *ATM^R189T^* mutation in the CLL patient two years before cth detection.

Moreover, *SETD2* deletions have been associated with the loss of *TP53*, genomic complexity, and cth and define a subgroup of patients with poor outcome (20). The published data highlight *SETD2* aberrations as a recurrent, clonal, early loss-of-function event in CLL pathobiology that appears to be the result of cth and linked to aggressive disease. In this comprehensive study, 26% CLL cases with *SETD2* deletions showed evidence of cth on chromosome 3, constituting predominantly cases with ultra-high-risk CLL. Another study also proved that *SETD2* inactivation is more frequent in CLL cases with cth than in non-cth cases (26% versus 1.4%) (38).

In the case study by Bassaganyas et al. (17), the authors found cth-derived deletion of 6q21 spanning the *NFKBIE* gene. In general, del(6q) is known to be present in 6% of CLL and linked to shorter progression-free survival (3, 46, 55). In the reported case, the concurrent *NFKBIE^E285X^* mutation on the other allele led to the absence of a functional *NFKBIE* in cth-subclone. Moreover, del(10q24) involving *NFKB2*, a subunit of NF-κB transcription factor complex regulating the *NFKBIE* transcription, was observed (17).

Although seen with low frequency, there were observations of the cth-related gain of 8q (the *C-MYC* gene) (19), loss of chromosome 13 (mir-15a/mir-16) (5, 38), and loss of 14q (16), which are recurrently detected in CLL. Loss of 8p, associated with a higher number of CNAs in CLL (56, 57), was also observed in cth-CLL (39). In addition, RNA-Seq revealed a fusion transcript of *UBR2-SPATS1* in one case (17) potentially contributing to disease aggressiveness, as the *UBR2* gene is involved in the cell growth controlling (58) and could have been deregulated or have gained a new function due to premature truncation and fusion with the second partner.

## Associations of Telomere Biology and Chromothripsis in CLL

Telomere dysfunction is known to have a dynamic role in shaping a disease course in CLL (59, 60). Physiological telomere shortening corresponding to the number of divisions a cell goes through leads to gradual uncapping of the chromosome ends. At a certain critical point, telomeres are recognized as DNA double-strand breaks and trigger the DDR. As a consequence, the senescence and/or apoptosis checkpoints are activated to prevent neoplastic transformation (61). If protective mechanisms are compromised, cells may continue to proliferate, which results in genomic instability (62). Studies have shown that CLL cells have a close inverse correlation between telomere length and telomerase activity compared to healthy cells (63–66). This could be explained by the theory that the genomic instability associated with shorter telomeres promotes the selection of fit CLL clones that overcome senescence and sustain cell survival due to the maintenance of minimal telomere length by telomerase. It was shown that the tumor microenvironment-mediated signaling, such as BCR or PI3K signaling, contributes to telomerase activation (67).

The dysfunctional telomeres often induce intra- or inter-chromosomal end fusions that can occur as clonal events. Their frequency was found to increase with the advancing disease stage in CLL (68). Such telomere fusions result in the formation of dicentric chromosomes that undergo breakage at the anaphase. This phenomenon is known as the breakage-fusion-bridge (BFB) cycle (69) and can be a precursor to genomic complexity such as cth (23, 70) (**Figure 1C**). Studies described the association of short telomeres with complex karyotypes (64, 71, 72) or with a higher number of CNAs (73, 74) in CLL. Unlike other tumor entities (e.g. central nervous system tumors), CLL cells were shown to have shorter telomeres in the cases with cth as compared to the cases without cth (70).

In general, the telomere length has been proposed to be an independent prognostic factor in CLL, with short telomeres being associated with adverse outcome (63–66, 73–76), the presence of del(11q) and del(17p) (64, 72–75, 77), as well as mutations in *ATM* and *TP53* (72–74, 76–78) both of which serve as critical checkpoint genes activated upon telomere shortening. However, the association between telomere dysfunction and cth was confirmed to be independent of the *TP53* mutation status in CLL (70). It has been supposed that in cases where no somatic *TP53* mutation was detected, other aberrations affecting the DDR and/or potentially inducing p53 dysfunction likely allow the cell to avoid apoptosis despite telomere dysfunction. On the other hand, del(17p) treatment-naïve CLL patients with cth have significantly shorter telomeres compared to those without cth (79). Moreover, loss of *SMC5*, which is involved in maintaining genomic stability and plays a role in telomere-related functions, might favor cth, especially when co-occurring with short telomeres and *TP53* defects (79). In addition, certain CLL cases with cth-like patterns in the 5p region were discovered, including gains of *TERT*, which encodes the telomerase reverse transcriptase (18).

It is presumed that the derivative chromosomes resulting from cth are likely stabilized hindering further progressive chromosomal cataclysm that would be incompatible with cell survival. From longitudinal observations, the chromothriptic patterns in CLL patients are either stable, in which case the relapse specimens show similar aberrations to the primary samples (5, 70), or they are lost by clonal selection in the relapse (17). Thus, telomere stabilization mechanisms are likely activated after the occurrence of cth to prevent continuing (and presumably lethal) genome-wide disruption.

All the mentioned findings confirm that the telomere attrition followed by end-to-end chromosome fusion and subsequent breakage leads to cth in CLL. This is followed by the establishment of telomere maintenance mechanisms that “lock-in” these alterations and prevent further lethal events. It, therefore, highlights the importance of detecting cth in the context of telomere length for risk stratification as well as for monitoring and early identification of clonal changes. Similarly, telomere maintenance mechanisms may represent a target for therapeutic intervention in cth-positive cases.

## Chromothripsis in CLL Diagnostics and Treatment

The available data suggest the potential of cth detection for better stratification of CLL patients by recognizing cases with highly complex karyotypes and thus adverse prognosis. Studies showed that cth-CLL patients show adverse clinical course and demand an early therapeutic intervention (2, 3, 5, 18, 19), often even evolving refractory disease (3, 5, 20).

As follows from the information above, cth is a consequence of genomic instability and is associated with aberrations in specific molecular pathways (15, 22, 80). In these cases (presumably more than in others) the cell signaling inhibitors should provide a promising outcome, similarly to the cases with defective *TP53*. However, no studies are available yet.

In general, the detection of cth-associated abnormalities could serve for the identification of molecular therapeutic targets. For instance, targeting oncogenes amplified *via* cth might provide a therapeutic benefit. Additionally, leukemic cells with cth could successfully respond to immune checkpoint blockade due to potential neoantigens generated from genomic rearrangements (81). The neoantigens were proven to bind patient-specific major histocompatibility complex molecules and to expand tumor-infiltrating T cell clones (82). These findings might be exploited for the development of novel immunotherapeutic approaches as well as the selection of patients to be administered immunotherapies. This strategy has already been suggested for a subset of AML patients with a high burden of alterations (32). Similarly, cth-derived fusion genes can help to increase the sensitivity of cancer cells to certain types of agents. An example is a patient with myelodysplastic syndrome, where several cryptic fusions, including *ETV6–PDGFRB*, were found (83). This is underlain by the fact that the myeloid neoplasms associated with *PDGFRB* rearrangement represent a specific entity sensitive to tyrosine kinase inhibitors (84).

Besides that, a synthetic lethality approach (85) is also an option for cth-CLL treatment. This approach is based on targeting a synthetic-lethal partner of a gene that is already mutated or overexpressed – that means targeting a gene that is compensating for the loss of activity of the dysfunctional one. Simultaneous inactivation of such gene pair results in cell death (85). As the defects in the DDR mechanism are frequently associated with cth, the cells have an increased level of DNA damage and evolve new mechanisms to resist endogenous and exogenous stress. The strategy of synthetic lethality in such cases could combine current treatment modalities with drugs targeting residual DNA repair pathways that such cells are dependent on (86).

## Conclusions

Based on the available data, cth is a recurrent event in CLL and could have a strong prognostic value. Although there is rapid progress in understanding molecular processes behind cth, current studies have important limitations. The biggest drawback is a relatively small number of CLL patients that have been analyzed so far which hampers the reproducibility of published results. Another issue is missing longitudinal observations. Most studies focus on a single time point of the disease, usually treatment-naïve. However, the information about the dynamics of the cth and the changes accompanying this event is lacking. It would be of interest to elucidate which changes precede the development of cth and which, in contrast, are more frequently its consequence. These findings would facilitate a better understanding of CLL clonal evolution and its driving forces and could reveal recurrently altered molecular pathways with different prognostic impacts.

The genomic landscape induced by cth is complex and linking cth to specific clinical outcomes is not always straightforward. The genes and genomic regions affected by cth appear to be the most important factors for the disease phenotype, not the occurrence of cth itself. This highlights the growing need for personalized medicine to be implemented into CLL treatment. Analyzing tumor samples at different time points should also be a part of the clinical program to elucidate clonal genotypes that could be therapy-resistant, which might help in therapeutic decisions along the disease course.

## Author Contributions

KZ drafted the manuscript and created figures. KP proposed the structure and supervised manuscript preparation, both authors performed the literature search and contributed to manuscript writing. Both authors contributed to the article and approved the submitted version.

## Funding

The authors acknowledge the support by the AZV project NU21-08-00237 and the program for the conceptual development of research organization FNBr 65269705 provided by the Ministry of Health of the Czech Republic, the student projects MUNI/A/1595/2020 and MUNI/IGA/1640/2020 provided by the Ministry of Education, Youth and Sports of the Czech Republic, and the European Regional Development Fund Project “A-C-G-T” No. CZ.02.1.01/0.0/0.0/16_026/0008448. KZ is a holder of Brno Ph.D. Talent 2019 Scholarship funded by the Brno City Municipality. The content of this manuscript is a part of the doctoral thesis of KZ.

## Conflict of Interest

The authors declare that the research was conducted in the absence of any commercial or financial relationships that could be construed as a potential conflict of interest.

## Publisher’s Note

All claims expressed in this article are solely those of the authors and do not necessarily represent those of their affiliated organizations, or those of the publisher, the editors and the reviewers. Any product that may be evaluated in this article, or claim that may be made by its manufacturer, is not guaranteed or endorsed by the publisher.
